# Successful Traceability of Wildlife Samples Contributes to Wildlife Conservation: A Case Study of Tracing the Snub-Nosed Monkey (*Rhinopithecus* spp.)

**DOI:** 10.3390/ani15020174

**Published:** 2025-01-10

**Authors:** Xibo Wang, Ying Shen, Yang Teng, Ruifeng Wu, Shuhao Liu, Jilai Zhao, Can Hu, Ming Li, Huijuan Pan, Jiwei Qi

**Affiliations:** 1 School of Ecology and Nature Conservation, Beijing Forestry University, Beijing 100083, China; wangxibo@bjfu.edu.cn (X.W.);; 2 CAS Key Laboratory of Animal Ecology and Conservation Biology, Institute of Zoology, Chinese Academy of Sciences, Beijing 100101, China; lim@ioz.ac.cn (M.L.);; 3 College of Life Sciences, University of Chinese Academy of Sciences, Beijing 100049, China; 4 School of Life Sciences, Hebei University, Baoding 071000, China; 5 Institute of Forensic Science, Ministry of Public Security, Beijing 100038, China

**Keywords:** wildlife trade, forensic identification, snub-nosed monkey, whole genome, geographical origin traceability

## Abstract

Rapid and effective methods for tracing the geographic origin of wildlife samples are essential for tackling the illegal wildlife trade. Traditional morphological categorization methods are often inadequate as relying on the mitochondrial COXI barcode is insufficient for determining geographic populations. To address these limitations, we developed a bioinformatics-based pipeline for the rapid identification of traceable nuclear genome loci. This pipeline has been applied to the whole-genome sequence (WGS) data of China’s flagship species, the snub-nosed monkey (*Rhinopithecus* spp.). These species are known for sex-biased dispersal and hybrid speciation, which complicates genealogy tracing. Using phylogenetic principles, we employed the Robinson and Foulds (RF) distance and scanned over 1,850,726 population-specific loci, identifying five pairs that can trace genealogy origins rapidly and cost-effectively using PCR. Additionally, we found that relying only on mitochondrial genetic information is insufficient for rapid and accurate traceability to subspecies-level geographic populations. Our pipeline efficiently identifies loci and traces the geographic origin of snub-nosed monkey individuals, providing a valuable tool for species preservation and combating the wildlife trade. This approach can be extended to other species, aiding in the conservation of endangered wildlife and tracing criminal evidence.

## Simple Summary

In the current context where illegal wildlife trade is rampant, we have endeavored to develop a sample tracing method applicable to most wildlife species. We based our approach on the whole-genome resequencing data of five species within the genus *Rhinopithecus*. The species within the *Rhinopithecus* genus are all precious and endangered, with the majority of their populations located in China. Historically, they have been widely threatened by the illegal wildlife trade. Using phylogenetic methods, we filtered samples to obtain a collection that represents the genetic characteristics of various geographical populations. Subsequently, we identified specific loci unique to each population and developed primers for them. We further validated the effectiveness of this method using samples from the *Rhinopithecus* genus in experimental verification. The use of PCR products for the identification of wildlife samples is cost-effective and convenient, meeting the tracing requirements for most wildlife species and serving the purpose of combating wildlife crime.

## 1. Introduction

The snub-nosed monkey genus (*Rhinopithecus* spp.) is one of the most endangered wildlife taxa in China. It comprises five species: *R. roxellana*, *R. brelichi*, *R. bieti*, *R. strykeri*, and *R. avunculus*, among which the first three are endemic to China. *Rhinopithecus* spp. were once widely distributed in Asia during the Pleistocene and Holocene [1]. Since the beginning of the nineteenth century, human population growth, environmental degradation, and hunting have led to a sharp decline in *Rhinopithecus* populations [2]. Presently, *R. roxellana* is distributed in three mountainous regions of China: Shennongjia (roxSNJ) in Hubei, Sichuan–Gansu (roxSG) (which includes two neighboring populations: Minshan, roxGSMS and Qionglai, roxQA), and Qinling (roxQL) in Shaanxi. The other four species are distributed in the following locations: *R. bieti* in Yunnan and Tibet, China, *R. brelichi* in Guizhou, China, *R. strykeri* along the border between China and Myanmar, and *R. avunculus* in northern Vietnam [3]. Today, snub-nosed monkeys are the most endangered and least genetically diverse non-human primates in the world [4]. A recent study of global genome-wide diversity in primates revealed that, among 233 species, *R. roxellana* had the lowest genetic diversity and heterozygosity [5]. In China, four of the five species of snub-nosed monkey are listed as first-class protected animals at the national level. At the international level, all five species are listed as endangered or critically endangered on the IUCN Red List. Snub-nosed monkey parts were considered to have valuable medicinal properties in ancient China, and owning them (as in the case of skin products) denoted a high social status [2]. Nowadays, these animals are sought after because of the ornamental value of their fur and poaching and hunting remain the primary threats to their survival [6]. In addition, a large number of snub-nosed monkey individuals exist today in captivity, and these individuals are often of unknown genealogy. A rapid and convenient method to trace snub-nosed monkey samples is urgently needed in order to combat the wildlife trade and to preserve these endangered populations.

The wildlife trade is accelerating the extinction of species worldwide and represents a threat to global biodiversity [7]. Despite nations now recognizing the need to combat the wildlife trade, the paradox of animal detection rates being lower than the poaching rates persists [8,9]. This has led to the absence of evidence in certain smuggling cases, preventing convictions. In addition, illegally traded wildlife species are often distributed in different provinces or administrative areas, meaning that the simple species identification may not be sufficient to solve smuggling cases, which would often require tracing the source of a sample to a particular area [10,11]. Thus, the accurate geographical traceability of traded species also plays an important role in the investigation of wildlife trade cases.

In the past, species were frequently identified and geographically traced based solely on morphological characteristics and geographical information. However, such an approach does not work for wildlife products and in cases where samples have been destroyed, morphological characters are missing, or no geographical information is available [9]. With the development of molecular technology, DNA identification techniques (for example, based on mtDNA) have been widely used in wildlife forensics [12]. In the early years, mtDNA technology became the dominant method to identify many rare and endangered species, such as elephants (Elephantidae), cats (Felidae), and canines (Canidae) [13,14,15,16]. However, the use of mtDNA still has some limitations, such as its fast evolutionary rate, nuclear copying, and introgression, which lead to inaccurate identification [17,18,19,20,21]. For example, in cases involving the identification of Asian closed-shelled turtle species of the *Cuora* genus, the results of analyses using both mtDNA and nuclear DNA (nDNA) showed inconsistent phylogenetic relationships because of interspecific hybridization, which led to the finding that nDNA data are more reliable and that using mtDNA alone may lead to incorrect identification [22]. With the development of genomics and high-throughput sequencing technologies, it became possible to trace species more accurately using nDNA [23]. Therefore, it is urgent to develop a method for the genetic tracing of wildlife based on whole-genome sequence (WGS) data that is inexpensive, accurate, and capable of tracing species at the species and population level.

In this study, we developed a polymerase chain reaction (PCR)-based method to geographically trace snub-nosed monkey samples based on WGS data obtained from 95 individuals. We designed multiple primer pairs for PCR analysis and experimentally validated their suitability. To select representative specific loci for each population more accurately, we first eliminated potentially related and mixed individuals based on population kinship analysis, gene flow assay, and population structure analysis. At the same time, we used the de novo complete mitochondrial genome to explore the possibility of tracing samples using mitochondrial sequence. We further searched for genes that conformed to the population differentiation based on phylogeny and finally scanned specific loci in these genes that could be used for traceability analysis. Our method can accurately trace *R. roxellana* samples, and the results here reported provide a basis for inferring the routes used to smuggle this species and thus potential evidence for the conviction of wildlife traffickers. This method can be used as an effective tool to protect *R. roxellana* and combat the wildlife trade. Our screening principles can be further extended to other species and provide scientific support for the conservation of other endangered wildlife threatened by poaching and for the traceability of criminal evidence.

## 2. Materials and Methods

### 2.1. Single Nucleotide Polymorphism (SNP) Genotyping

**Figure 1:**
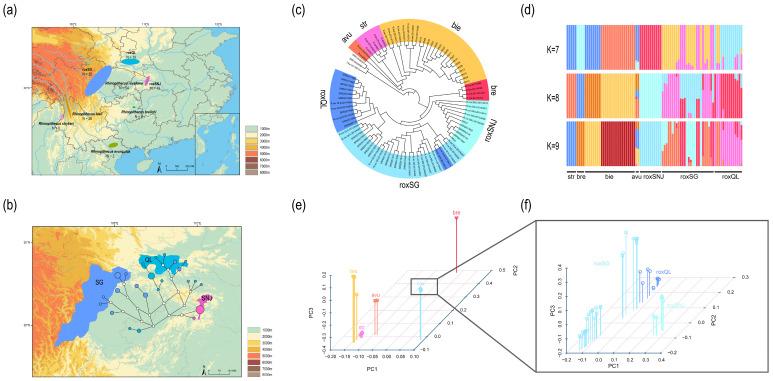
Number and distribution of snub-nosed monkey samples. (**a**) Haplotype network of the three *R. roxellana* populations based on the mitochondrial genome. (**b**) The ML tree (**c**) suggests that two roxQL and eight roxSG individuals were generated by hybridization. The results of admixture analyses (**d**) support the phylogeny of the snub-nosed monkeys and the gene flow within *R. roxellana* populations. The PCA results obtained for the five species (**e**) and *R. roxellana* (**f**) are consistent with the ML tree.

Illumina WGS data previously obtained from 95 snub-nosed monkeys belonging to five different species (54 *R. roxellana*, 28 *R. bieti*, 5 *R. brelichi*, 6 *R. strykeri*, and 2 *R. avunculus*) were used in this study to build a snub-nosed monkey traceability system [4,24,25,26] (Figure 1a; Table S1). To ensure the reliability of the traceability results, we utilized standard wild samples with clear geographical origins from the aforementioned studies, which accurately represent the actual genetic lineage of their respective populations. The read data were downloaded from the NCBI Sequence Read Archive public database. Additionally, genetic data for three langur species, i.e., Francois’s langur (*Trachypithecus francoisi*, SRR7778908), Laotian langur (*Trachypithecus laotum*, SRR10028098), and Javan langur (*Trachypithecus auratus*, SRR10028097), were downloaded from the NCBI database as outgroups to be used for the phylogenetic analyses [27].

The paired-end reads for all the samples were mapped to the *R. roxellana* reference genome (ASM756505v1, GCA_007565055.1) using the Burrows–Wheeler Aligner mem software v0.7.17 [28].

We performed SNP calling based on bam files with the Genome Analysis Toolkit (GATK, version 4.1.9) [29]. A GVCF file was obtained for each individual using the “HaplotypeCaller” method in GATK and then the samples were merged based on “CombineGVCFs”. Next, the “SelectVariants” function was applied to exclude indels and split variant and nonvariant loci, filtering the latter in subsequent analyses. The hard filter command “VariantFiltration” was applied to exclude potential false-positive variant calls using the following criteria: depth (QD) < 2.0, mapping quality (MQ) < 40.0, Fisher Strand (FS) > 60.0, HaplotypeScore > 13.0, MQRankSum < −12.5, and ReadPosRankSum < −8.0. To perform population structure analysis, high-confidence bi-allelic SNPs with less than 10% missing genotypes were screened using VCFtools (v0.1.15) [30], and the following criteria were met: “–max-missing 0.9–minQ 20–min-alleles 2–max-alleles 2”. SnpEff v4.3 [31] was used to annotate the SNPs based on the reference annotation of *R. roxellana* (GCF_007565055.1_ASM756505v1_genomic) (Table S2).

### 2.2. Filtering of Potentially Related and Mixed Individuals

The WGS data used in this study were obtained from various investigations conducted in different years, which meant that duplicate samples may have been present. To eliminate closely related individuals within a population, the relatedness of all samples in each population was evaluated using KING v2.2.5 (with default parameters) [32]. Specifically, the “-kinship” command in KING v.2.2.5 was used to estimate the kinship coefficient, which shows the percentage of SNPs in each individual that are in the same state (IBS0, identity by state zero). Closely related individuals were identified as those in pairs (twins and first-degree relationships) with a kinship coefficient over 0.354. These consanguineous individuals were excluded in subsequent analyses. Sex-biased dispersal has been reported within *R. roxellana*, with small female or male groups undertaking migration [24]. To obtain suitable genetic markers representing geographic populations, potentially mixed individuals containing genetic markers from other populations were removed based on WGS data. To identify hybrid individuals among the three populations examined, the gene flow among *R. roxellana* individuals was calculated using Dsuite [33]. Eventually, Rb18, Rb19, R.bie-2011A091, R.bre6574, R.rox_QLS-2011A074, SRR2017694, R.rox_SNJ-2013A022, R.str03076575, SRR2017665, SRR2017666, SRR2017667, SRR2017673, SRR2017676, SRR2017664, R.rox_QLA-2012A005, R.rox_QLA-2013A007, R.rox_QLN-2013A014, and R.rox_QLN-2013A015 were removed in subsequent scans.

### 2.3. Mitochondrial Genome Assembly and Network Construction

Amplification of mitochondrial genes is nowadays the most commonly used method for species identification, and it has a promising ability to discriminate between vertebrate species [34]. In order to explore its discriminatory ability at the population level of *R. roxellana*, MitoZ [35] was used for the de novo assembly of the mitochondrial genome sequence of each *R. roxellana* individual from raw reads. For the mitochondrial genomes that were not circular, NOVOPlasty v4.3.1 [36] with K-mer set to 33 was used to assemble the mitochondrial genome, while the mitochondrial genes of a circularity individual were used as seed and reference. To assess the feasibility of using mitochondrial sequences to accurately trace snub-nosed monkey samples, mitochondrial haplotype networks were constructed using fastHaN [37] after matching the mitochondrial genomic data in MEGA v11.0.13. Finally, the results were visualized using the online website tcsBU (https://www.fc.up.pt/pessoas/amsantos/tcsBU/, accessed on 1 May 2024) [38].

### 2.4. Population Structure and Admixture Analyses

Population structure and phylogenetic relationships were examined using a maximum likelihood (ML) tree built with RAxML v8.2.11 [39] based on the whole-genome SNP set [minor allele frequency (MAF) ≥ 0.05]. The online software iTOL v7.0 (https://itol.embl.de/, accessed on 20 May 2024) was used to visualize the results [40]. Principal component analysis (PCA) was performed for two datasets (i.e., dataset 1, all snub-nosed monkey individuals; dataset 2, all *R. roxellana* individuals) using VCF2PCACluster v1.40. Then, ADMIXTURE v1.3.0 [41] was used to investigate the ancestral populations, with the analysis being run 100 times for each K with coancestry clusters ranging from 2 to 9. The best K value was estimated using a cross-validation procedure in admixture.

The fineSTRUCTURE v4.1.1 [42] uses information about the relative positions of the mutations in the genome. Therefore, it was used along with chromoPainter to find patterns of haplotype similarity based on much of the information present in the haplotype structure to obtain a fine-scale population structure [43].

### 2.5. Scanning of Whole-Genome Specific Loci and Primer Design

**Figure 2:**
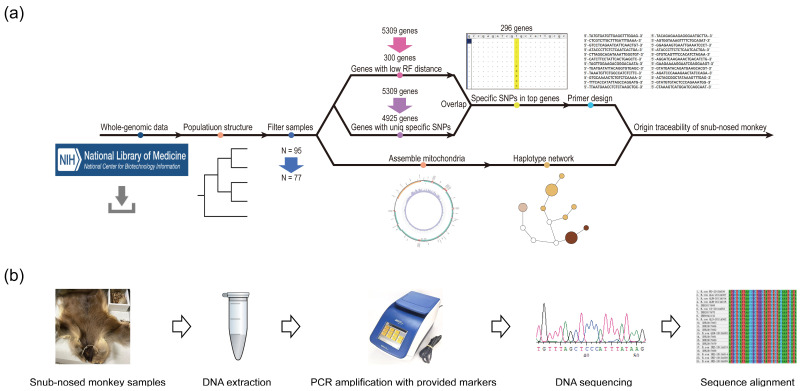
Pipeline of the traceability procedure applied to the snub-nosed monkey samples. (**a**) Traceability procedure using the primers designed in this study. (**b**) After DNA extraction, PCR, DNA sequencing, and sequence alignment, it was possible to determine the samples’ origin based on specific amplified loci.

To design specific molecular markers, the selected gene regions must be sufficiently long; therefore, genes with lengths > 2000 bp were initially screened. The Robinson and Foulds (RF) distance is the topological distance between two trees [44]. In this study, the RF distance between each gene tree and species tree was calculated and, the smaller the distance, the more similar the trees were. RAxML and the “–rfdist” command were used to build the gene trees and determine the RF distance for each of them, respectively. Correlation analysis between gene length and RF distance was carried out using the R package “ggplot” [45]. To identify genes suitable for the design of specific primers, we sorted all genes based on their length and RF distance. Ultimately, we selected genes that are genetically close to the phylogeny of the snub-nosed monkeys and have appropriate lengths to design primers as our target genes (Figure 2a).

For every snub-nosed monkey population, the variant loci were extracted using our internal script (https://github.com/DRWTSON/Wildlife-specific-loci-scanning/tree/main, accessed on 4 December 2024), and the R package “UpsetR” [46] was used to count them and determine their distribution. Based on the variant loci, the population-specific loci were then extracted, and their positional information was combined to identify those located in the target genes. RectChr (https://github.com/BGI-shenzhen/RectChr, accessed on 8 June 2024) was used to visualize the specific loci and inspect gene positions. Through visual inspection, the positions of the specific loci were determined and the target sequences were finally imported into MEGA11 [47] to examine the locus-specific information, which was visualized in Jalview [48].

A sequence of about 1800 bp near the target loci (900 bp on each side) was extracted using SAMtools v1.7 [49] and the target sequences were imported into the online website Primer3 [50] (https://primer3.ut.ee/, accessed on 17 July 2024). The parameters were set to: primer size, min 20, opt 22, and max 26; primer Tm, min 52, opt 56, and max 62; primer GC%, min 30, opt 50, and max 70; and product size ranges 850–950. For every snub-nosed monkey population examined in this study, multiple sets of primers were created to guarantee the validity of the findings.

### 2.6. DNA Extraction and PCR Validation

Single-blind experiments were performed using muscle or blood samples from known sources and the primers were validated. The primers were validated using available muscle or blood samples obtained from eight *R. roxellana* (four from roxSNJ, two from roxSG, two from roxQL), two *R. brelichi*, one *R. bieti*, and two *R. strykeri* individuals stored at −80 °C. *R. avunculus* samples were not available due to its critically endangered status. Total genomic DNA was extracted from the samples using the CTAB and phenol/chloroform methods [51].

The primers designed in this study were used to amplify the snub-nosed monkey DNA via PCR. The reaction was conducted in a total volume of 25 μL containing 2 × Es Taq MasterMix (Dye) (with Es Taq DNA polymerase), 3 mM MgCl_2_, and each 2′-deoxynucleotide triphosphate (dNTP) at a concentration of 400 μM. The following quantities were used: 12.5 μL of Taq MasterMix, 2 μL of DNA template, 1 μL of each forward and reverse primer (10 ng/μL), and 8.5 μL of ddH_2_O. The gap at the primer position was joined under the same PCR conditions (i.e., denaturation at 94 °C, annealing at 52 °C, and extension at 72 °C). After gel electrophoresis, suitable primers were selected for application to the snub-nosed monkey samples for traceability analysis.

## 3. Results

### 3.1. Variant Calling and Filtering of Samples Derived from Potentially Mixed Individuals

A total of ninety-eight individuals consisting of ninety-five snub-nosed monkeys and three individuals belonging to outgroups (*Trachypithecus francoisi*, *Trachypithecus laotum*, and *Trachypithecus auratus*) were examined in this study. The WGS reads obtained from each individual were mapped to the reference genome [52] using bcftools. An average mapping rate of 98.30% (78.08–100%), sequencing depth of 13.74 (7.56–51.23), and coverage of 97.04% (85.27–98.49%) were observed for the 98 individuals examined.

A total of eight potential admixed individuals were removed (estimated kinship coefficient > 0.3536) based on kinship analysis (Figure S1 and Table S3). After rigorous quality control and filtering, eight out of the ninety-eight individuals were eliminated, and 17,365,517 SNPs were retained from 134,948,888 SNPs for further analysis.

According to the results obtained from species trees, two roxQL individuals were divided onto roxSG branches, which was in line with the findings of the admixture analysis. A total of eight roxSG individuals (R.rox_QLA-2012A005–SRR2017667) were also shown to share a branch with roxQL (Figure 1c). In the admixture analysis, K = 8 showed the lowest cross-validation error (Figure 1d and Figure S2). For K = 2, *R. roxellana* was divided into two clusters with other snub-nosed monkey species. For K = 4, most of the *R. roxellana*, *R. brelichi*, and *R. strykeri* individuals as well as some *R. bieti* individuals were completely separated. For K = 8, all five species resulted separated, with roxSNJ being clearly distinguished from roxSG and roxQL within *R. roxellana*.

As expected, the PCA results were in line with those obtained from the phylogenetic tree (Figure 1e,f). For all individuals, the first principal component separated *R. brelichi*, *R. avunculus*, and *R. roxellana* from each other (PC1 = 50.17%), and the third eigenvector separated *R. strykeri* from *R. bieti* (PC3 = 7.04%). For *R. roxellana*, the first eigenvector separated roxSNJ from other populations (PC1 = 17.82%). Eight individuals from roxSG were clustered into roxQL in the second eigenvector (PC2 = 12.99%), and four individuals from roxQL were clustered into roxSG in the third eigenvector (PC3 = 5.90%).

The fineSTRUCTURE analysis identify relationships between individuals based on their most recent coancestry revealed that some locations were supported as distinct genetic populations (Figure S3). Gene flow analysis further explored hybridization of potentially mixed individuals. The presence of hybrid individuals, screened via population structure analysis, indicates gene flow between populations (Figure S4).

### 3.2. Mitochondrial Genome Assembly and Network Construction

The mitochondrial genomes of all *R. roxellana* individuals were assembled to test whether such a complete genome dataset could be used to discriminate the geographical origin of samples. The circular mitochondrial genomes of all 51 *R. roxellana* individuals (11 roxSNJ, 26 roxSG, and 14 roxQL individuals) were obtained. By constructing the haplotype network, a total of twenty-four haplotypes were identified (two for roxSNJ, thirteen for roxSG, and nine for roxQL individuals) (Figure 1b).

According to the haplotype network diagram, the roxSNJ population was relatively independent and was maternally differentiated from the other two populations. In contrast, in both populations roxQL and roxSG, haplotypes partially derived from each other were observed. Therefore, only roxSNJ individuals could be distinguished based on the mitochondrial genome, and the snub-nosed monkey samples could not be accurately traced.

### 3.3. Scanning of Specific Loci of nDNA

**Figure 3:**
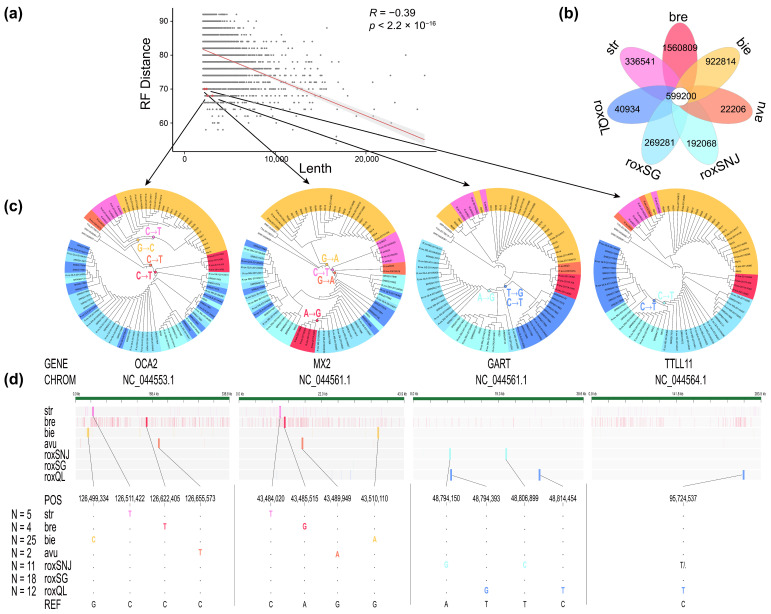
RF distance (similarity between the gene tree and the species tree) decreases as the gene length increases. (**a**) We screened specific loci from the intersection of fixed loci of the five snub-nosed monkey species. (**b**) Four genes with small RF distances and short lengths were selected to construct gene trees using the ML method. (**c**) The SNP variant loci in the genome were scanned and (**d**) the specific loci located on the four target genes were obtained for each population.

Firstly, all the fixed loci in the species examined, i.e., 336,541 for *R. strykeri*, 1,560,809 for *R. brelichi*, 922,814 for *R. bieti*, 22,206 for *R. avunculus*, 192,068 for roxSNJ, 269,281 for roxSG, and 40,934 for roxQL, were filtered out (Figure 3b). The results of population structure analysis showed that it was extremely difficult to directly distinguish roxSG and roxQL in *R. roxellana*. Eventually, after filtering, a total of 235,665 specific loci for *R. strykeri*, 1,503,165 for *R. brelichi*, 67,723 for *R. bieti*, 22,038 for *R. avunculus*, 3567 for roxSNJ, 88 for roxSG, and 126 for roxQL were identified based on loci fixed in all individuals.

The exon regions of genes are more strongly constrained by selection than the intronic and intergenic regions, indicating that they are more susceptible to natural selection during evolution because exons encode functional parts of proteins that are essential for the survival and reproduction of organisms [53]. In this study, variation in gene regions was prioritized, as these are the loci where stable genetic differentiation takes place, and therefore their loci were screened. First, exons shorter than 15 bp were excluded, and the retained ones were then screened for genes longer than 2000 bp (5309 genes) in order to be able to amplify the target region. The results of correlation analysis showed a significant negative correlation between the RF distance and gene length for 5309 genes (R = −0.39, *p* < 2.2 × 10^−16^) and, based on these two parameters, 300 target genes were selected (Figure 3a).

The genes in which the specific loci are located were counted, and the specific loci were present on 296 of the 300 target genes. It was found that each snub-nosed monkey species could be distinguished by two SNPs, which were NC_044553.1 (position: 126,511,422 bp, C-T) and NC_044561.1 (position: 43,484,020 bp, C-T) for *R. strykeri*, NC_044553.1 (position: 126,622,405 bp, C-T) and NC_044561.1 (position: 43,484,515 bp, A-G) for *R. brelichi*, NC_044553.1 (position: 126,499,334 bp, G-C) and NC_044561.1 (position: 43,510,110 bp, G-A) for *R. bieti*, and NC_044553.1 (position: 126,655,573 bp, C-T) and NC_044561.1 (position: 43,489,949 bp, G-A) for *R. avunculus* (Figure 3c). Within *R. roxellana*, the roxSNJ population could be distinguished by two SNPs, i.e., NC_044561.1 (position: 48,794,150 bp, A-G) and NC_044561.1 (position: 48,806,899 bp, T-C), and the roxQL population could be distinguished by three SNPs, i.e., NC_044561.1 (position: 48,794,393 bp, T-G), NC_044561.1 (position: 48,814,454 bp, C-T), and NC_044564.1 (position: 95,724,537 bp, C-T) (Figure 3c). These SNPs were tested for their potential use as traceable loci.

### 3.4. Traceability Analysis and Primer Validation

**Figure 4:**
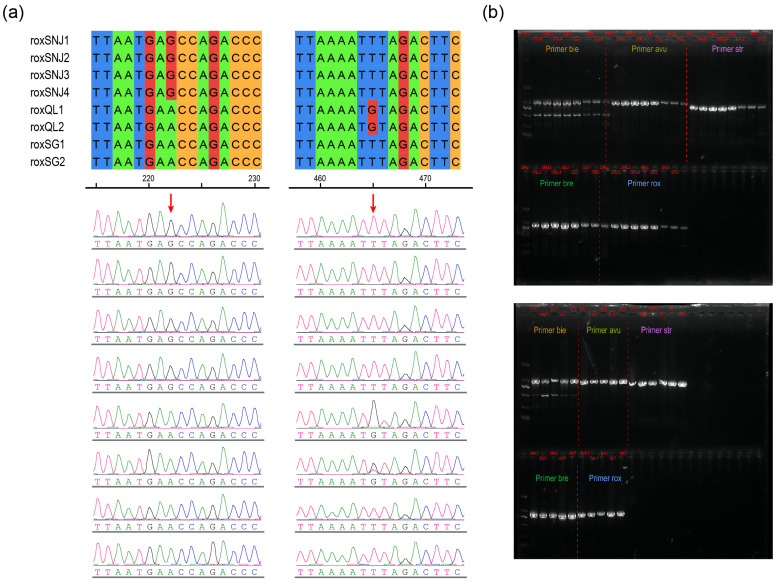
Results of the PCR amplification of *R. roxellana* samples using the primers designed in this study. (**a**) Four individuals from roxSNJ had a G base at 222 bp and the rest had an A base. Two individuals from roxQL had a G base at 465 bp and the rest had a T base. Two individuals from roxSG had an A base at 222 bp and a T base at 465 bp. (**b**) The PCR products obtained from snub-nosed monkey DNA samples from six geographic populations were subjected to gel electrophoresis and produced unique bands for all samples.

To allow the amplification of specific loci by PCR at a low cost, the primers were designed based on the screened target genes. Based on the results of multiple nucleotide sequence alignments, we selected a few segments of sequence containing specific loci for each species (these specific loci are located on four genes: *OCA2*, *MX2*, *GART*, and *TTLL11*). To ensure the reliability of the results, multiple sets of primers were designed for each population. These primers were used in single-blind experiments on golden monkey DNA samples obtained from a number of known sources. Then, five SNP-based molecular markers were screened using multiple pairs of designed primers to ensure that the primers provided in this study are all suitable (Table 1). The results showed that the target sequences of four individuals from roxSNJ, two individuals from roxQL, two individuals from roxSG, two individuals from *R. bieti*, one individual from *R. brelichi*, and two individuals from *R. strykeri*, respectively, were successfully amplified and that all the amplified specific loci were also matched to each population (Figure 4a). Furthermore, the gel electrophoresis analysis of all PCR products produced clear bands, indicating that the target specific sequences of each snub-nosed monkey population could be amplified using these primers (Figure 4b). All five sets of specific primers screened showed 100% traceability reliability in single-blind experiments and can be applied to actual wildlife cases.

## 4. Discussion

### 4.1. Mitochondrial DNA Provides Low Resolution for Population Level Traceability of R. roxellana

At present, wildlife forensic cases are difficult to solve due to the degraded or limited samples available for analysis [9]. MtDNA is more resilient to degradation and is present in larger copy numbers, and short mtDNA fragments containing effective loci in severely damaged samples can be easily amplified [54]. Therefore, this DNA type is widely used for the identification of various species and their origin [13,15,55]. Haplotype networks based on mtDNA can represent intraspecific genetic variation, making mtDNA-based identification a common method for describing phylogenies and developmental patterns [56]. In addition, it is possible to visualize sample distributions and identify distinct lineage populations using mtDNA [57]. Thus, it has been suggested that mitochondrial genome data allow one to obtain haplotype networks with a sufficient resolution to resolve issues concerning the biogeography of populations [58]. However, according to our study, in *R. roxellana*, the mtDNA does not have the ability to discriminate at the population level due to migration and hybridization between geographic populations. In this study, due to the sex-based dispersal of *R. roxellana*, the mtDNA does not have the ability to discriminate at the population level.

### 4.2. Applying WGS Data for Traceability

Complex historical dynamics, extensive sex-biased dispersal, and hybridization indicate a complex flow among *Rhinopithecus* species or populations [24,26]. Thus, in this study, we first filtered out snub-nosed monkey individuals exhibiting potential introgression signals and close kinship and we have then conducted fine-scale population structure and gene flow analyses to further verify hybrid individuals. Compared to common methods, such as PCA and admixture analysis, the fineSTRUCTURE v2.0 software can explicitly model the correlation between neighboring SNPs due to linkage disequilibrium and use extended multi-marker haplotypes distributed throughout the genome [42]. In addition, a statistical approach based on Patterson’s D, also known as ABBA-BABA, can be used to assess gene flow among individuals. Even if there is gene flow within the snub-nosed monkey populations, drift or selection in different geographic populations during prolonged isolation and local adaptation can cause mutations to accumulate differently in them, leading to specific loci in some genetic groups. These loci are not always the result of adaptive selection and may also be generated via random fixation by drift [59,60]. Therefore, among individuals in different geographic regions, there should be specific SNP loci that can be amplified to determine the geographic origin or even the population to which a sample belongs, making these markers effective for wildlife traceability analyses [61].

In this study, we visually screened for potentially mixed individuals that were phylogenetically interspersed with other genetic lineages and retained representative individuals from each geographical population. The results of the gene flow test for these admixed individuals indicated significant gene flow from other populations (Table S4). Even after strict filtering, we also found that it was still possible to detect specific loci for each of the snub-nosed monkey species and each *R. roxellana* population using WGS data (Figure 3d), although genus *Rhinopithecus* has a very low genetic diversity [5]. In addition, a recent study revealed that *R. brelichi* derived from hybridization between *R. roxellana* and the ancestor of *R. bieti*/*R. strykeri* and that, in the ancestor of *R. brelichi*, the genes of parent A (*R. roxellana*) and parent B (ancestor of *R. bieti*/*R. strykeri*) showed a mosaic distribution [62], which may greatly increase the difficulty of tracing geographic origins using a small number of single loci. However, some of these loci have been fixed during their independent evolution as population-specific loci; thus, hybrid genomes may stabilize at the genome level due to geographical isolation following founder events [63]. In the present study, after integrating all the snub-nosed monkey WGS data, we filtered 1,560,809 independent fixed loci in *R. brelichi* and retained 37,926 specific loci within 297 target genes (Figure 3b). Subsequent verification via PCR (Figure 4b) indicated that these specific loci filtered using our traceability method could effectively represent the isolated lineage of *R. brelichi*.

In short, analysis using mitochondrial genome data alone was not sufficient to differentiate the roxSNJ, roxSG, and roxQL populations (Figure 1b), and samples could not be accurately traced at the population level. However, successful tracing could be achieved at both the population and species levels by applying our method to WGS data (Figure 2a). These results further highlight the invaluable advantages of WGS data in terms of precision over the DNA barcoding based on mitochondrial data in wildlife forensics.

## 5. Conclusions

We provided a valid method for accurately identifying snub-nosed monkey samples of unknown origin (Figure 2b). Snub-nosed monkeys are the primates with the lowest heterozygosity levels in the world and exhibit extensive sex-biased dispersal, implying that both mitochondrial and nuclear genes introgression in their populations. With the advent of next-generation sequencing technology, sufficient amounts of WGS data and variant loci have become available for wildlife traceability analyses at the population level. Given the limited number of hybrid individuals present, misidentification is inevitable. Furthermore, the PCR-based methods for tracing wildlife samples are rendered ineffective when dealing with samples that have undergone excessive tanning and high-temperature processing. However, for the illegal trade in snub-nosed monkeys, the findings of our study can still deduce the geographical origins of the majority of samples, and so we hope that our approach can effectively complement precise population-level tracing under the premise of insufficient mitochondrial accuracy. By combining genome-based methods with other traceability techniques, such as those based on gut microbes, isotopes, etc., the accuracy of tracing snub-nosed monkey samples can be further improved [64,65,66].

In summary, this research developed an accurate geographic traceability procedure that will be useful for identifying snub-nosed monkey species and populations from unidentified samples (e.g., skin, hair, carcass material, etc.). Five snub-nosed monkey species and three *R. roxellana* populations were successfully identified in this study. Our method can significantly narrow down the geographic origin of samples in actual wildlife illegal trade cases and assist in locating trading routes. Our method also provides a useful tool for the identification of snub-nosed monkey samples, allowing for the production of powerful evidence in smuggling cases and therefore contributing to enforcing wildlife protection laws and combating wildlife crime. At a time when animal populations are under threat, our study provides a paradigm for the development of accurate traceability methods that can be applied to other wildlife, which will contribute to mitigating the risk of species extinction.

## Figures and Tables

**Table 1:** The list of primers used for the amplification of four target genes via PCR. Using these primers, specific loci were amplified to trace the geographical origin of the snub-nosed monkey samples.

Population	Gene	Forward	Reverse	Amplicon Length
str	OCA2	5′-CTCGTCTTGCTTTGATTTGAAA-3′	5′-AGTGGTAAAGTTTTCTGCAGAT-3′	817 bp
bre	OCA2	5′-GTCCTCAGAATCATTCAACTGT-3′	5′-GGAGAAGTGAATTGAAATCCCT-3′	865 bp
bie	OCA2	5′-TTCCTCCATCCTCTAGTATTCC-3′	5′-ACAACAACAACAAAAGTAACCC-3′	972 bp
avu	MX2	5′-TAGTTGGAAGACGGGACAATA-3′	5′-GAAGAAAAGGAATCGAGGAAGT-3′	882 bp
rox	GART	5′-CTGGGCATCTGAAAATAGACAT-3′	5′-GTGTGTGTGTATATAGGCCTTT-3′	878 bp

## Data Availability

The data that support the findings of this study are openly available in the NCBI Sequence Read Archive at https://www.ncbi.nlm.nih.gov/, accessed on 15 February 2024, reference number: PRJNA271514, PRJNA230020, PRJNA247935, PRJNA261768, PRJNA283338, PRJNA448482, PRJNA616055, SRR10028097, SRR10028098, SRR7778908.

## Supplementary Materials

The following supporting information can be downloaded at: https://www.mdpi.com/article/10.3390/ani15020174/s1, Figure S1. Results of kinship analysis, individuals above the dotted line “duplicate/MZ twin” are considered repeated sampling. Figure S2. The Cross-validation (CV) error for varying values of K in the admixture analysis. Minimum of estimated CV error on K = 8 suggests the most suitable number of populations. Figure S3. Coancestry heatmap of fineSTRUCTURE unlinked model. The black lines indicate individuals belonging to each geographic group, and the black dashed lines mark the presence of inbred individuals within the golden snub-nosed monkey. The scale shows lower (yellow) to higher (blue) amount of shared genetic chunks between the inbred lines. Figure S4. Gene flow among individuals of golden snub-nosed monkeys was calculated using a method based on Patterson’s D (ABBA-BABA statistic). The legend shows the gene flow between two individuals: lower (blue) and higher (red), and the calculated *p*-value: lower (dark) and higher (light). Table S1. Sample information and sequencing data summary of Rhinopithecus individuals and outgroups used in this study. Table S2. Distribution of the number of effects within various genomic regions of snub-nosed monkeys. Table S3. Combinations of individuals with estimated kinship coefficients over 0.354 from the results of kinship analyses, these pairs of individuals considered to be duplicate or MZ twin. Table S4. Using the ABBA-BABA analysis to calculate gene flow for potential admixed individuals (P3) filtered out through phylogenetic results.
