# Supplementary material for: Successful Traceability of Wildlife Samples Contributes to Wildlife Conservation: A Case Study of Tracing the Snub-Nosed Monkey (Rhinopithecus spp.)
Source: Animals (Basel). 2025 Jan 10;15(2):174. doi: 10.3390/ani15020174 (PMC11758607; doi:10.3390/ani15020174)
Supplement: Supplementary file 1 [file animals-15-00174-s001.zip › animals-3383055-supplementary.pdf]

***Title*****:** Successful Traceability of Wildlife Samples Contributes to Wildlife Conservation: A Case Study Of Tracing Snub-Nosed Monkey (*Rhinopithecus* Spp.)

# Supplementary Materials

## Supplementary Figures

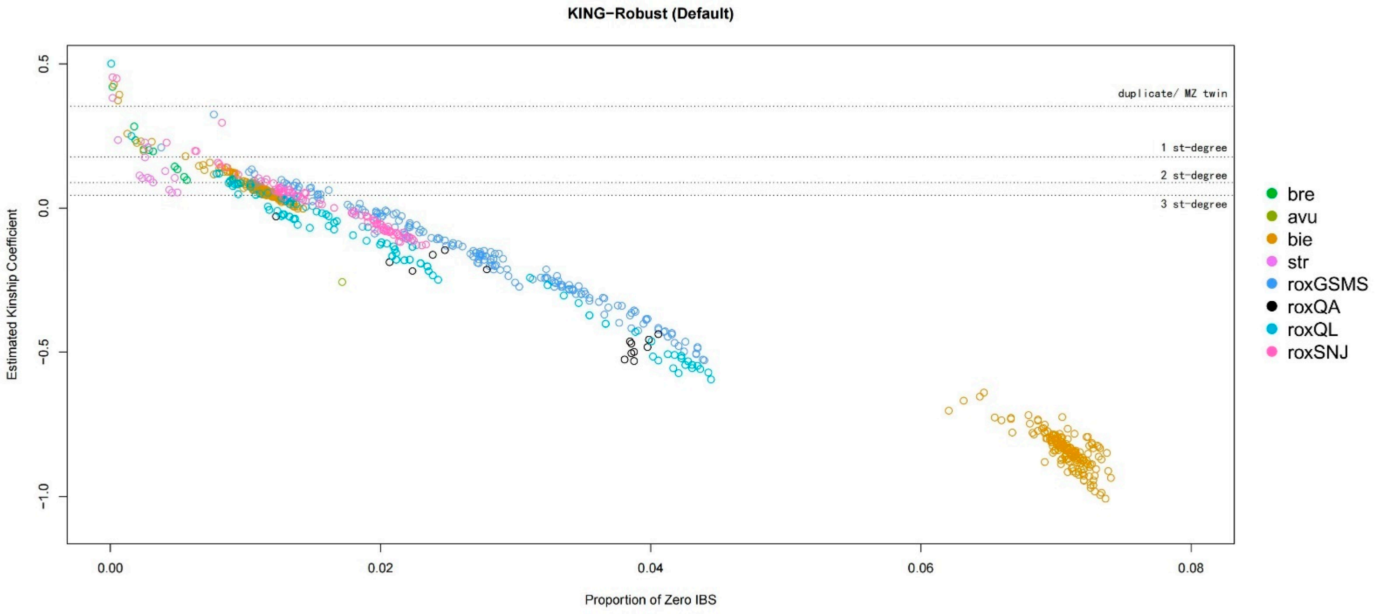


**Figure S1.** Results of kinship analysis, individuals above the dotted line "duplicate/MZ twin" are considered repeated sampling.

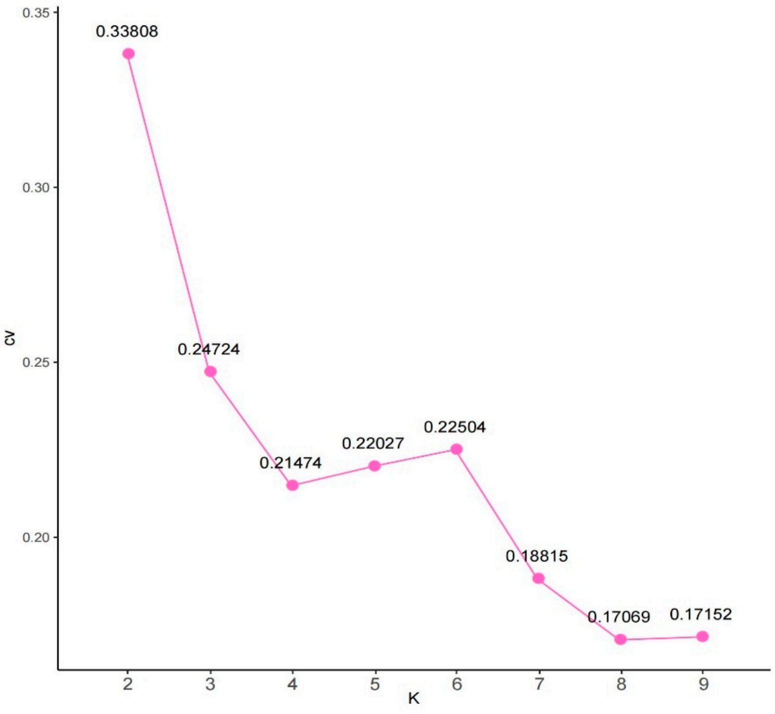


**Figure S2.** The Cross-validation (CV) error for varying values of K in the admixture analysis. Minimum of estimated CV error on K= 8 suggests the most suitable number of populations.

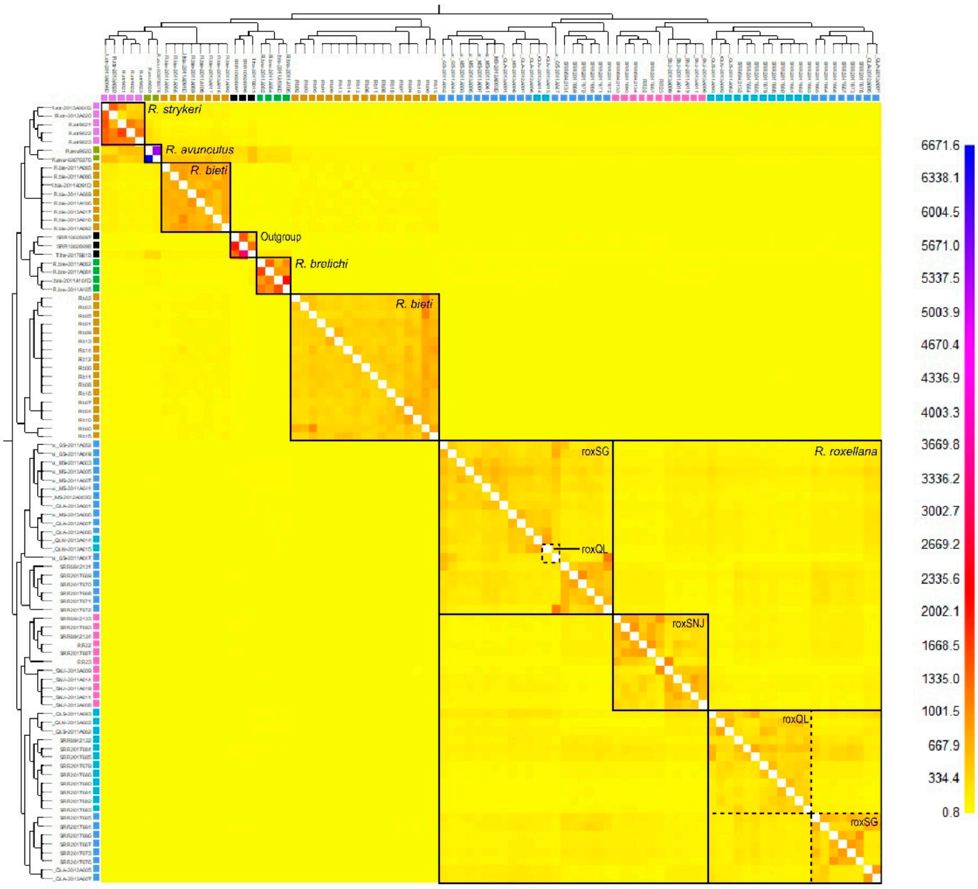


**Figure S3.** Coancestry heatmap of fineSTRUCTURE unlinked model. The black lines indicate individuals belonging to each geographic group, and the black dashed lines mark the presence of inbred individuals within the golden snub-nosed monkey. The scale shows lower (yellow) to higher (blue) amount of shared genetic chunks between the inbred lines.

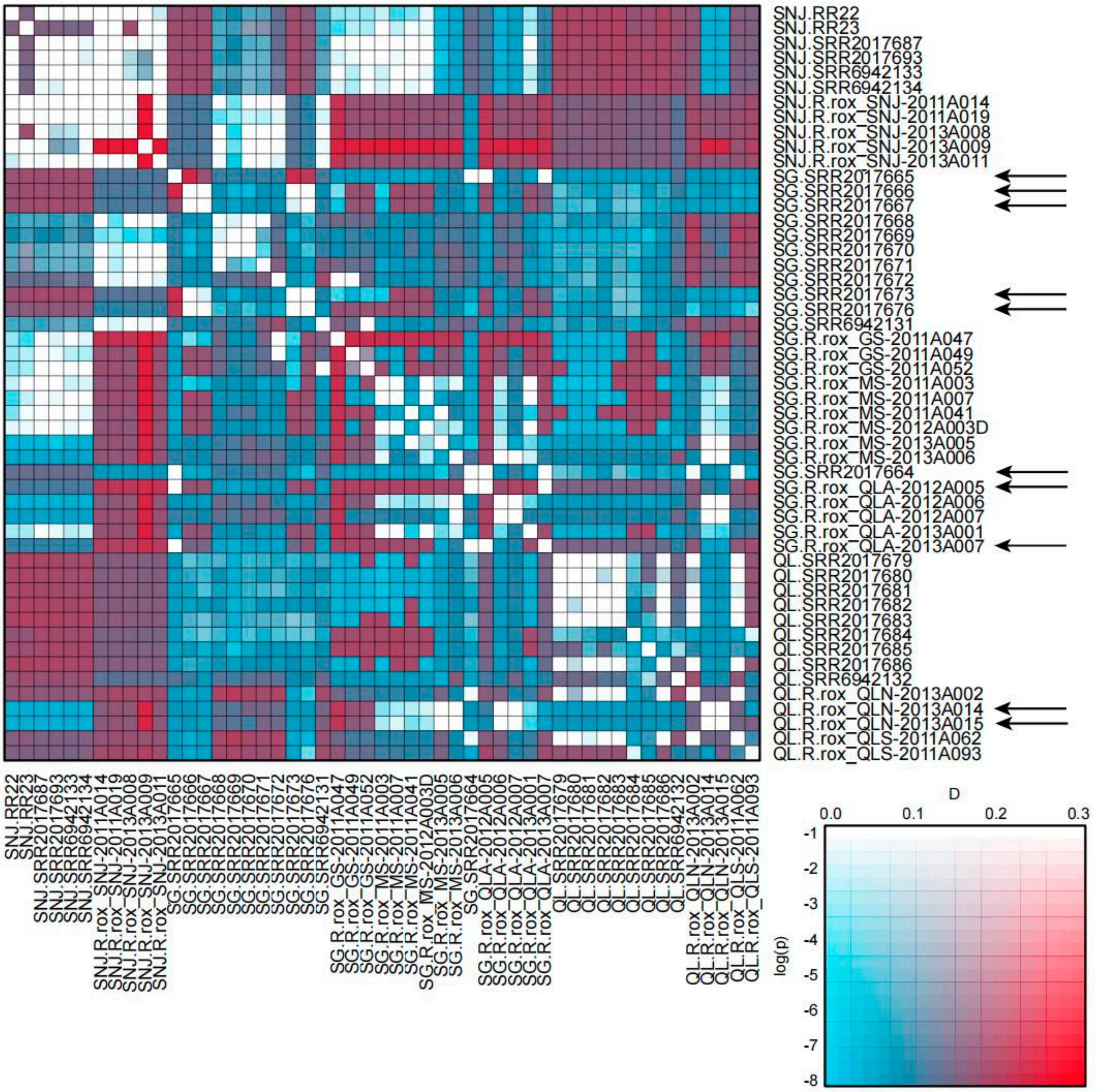


**Figure S4.** Gene flow among individuals of golden snub-nosed monkeys was calculated using a method based on Patterson's D (ABBA-BABA statistic). The legend shows the gene flow between two individuals: lower (blue) and higher (red), and the calculated p-value: lower (dark) and higher (light).

## Supplementary Tables

**Table S1.** Sample information and sequencing data summary of *Rhinopithecus* individuals and outgroups used in this study.

| Sample ID | Average sequencing depth | Coverage(%) | Mapping rate (%) | SRA Run ID | Species (Group) | Location | Reference |
|---|---|---|---|---|---|---|---|
| R.bie-2011A085 | 10.5 | 96.64 | 99.26 | SRR1739912<br>SRR1739913<br>SRR1739914<br>SRR1739915<br>SRR1739916 | *R. bieti* | Weixi, Yunnan Province, China | Zhou.2016 |
| R.bie-2011A086 | 10.74 | 96.78 | 99.26 | SRR1739927<br>SRR1739928<br>SRR1739929<br>SRR1739930 | *R. bieti* | Weixi, Yunnan Province, China | Zhou.2016 |
| R.bie-2011A089 | 9.25 | 96.91 | 98.93 | SRR1739931<br>SRR1739932<br>SRR1739933 | *R. bieti* | Weixi, Yunnan Province, China | Zhou.2016 |
| R.bie-2011A091 | 10.07 | 96.93 | 99.14 | SRR1739991<br>SRR1739992<br>SRR1739993 | *R. bieti* | Weixi, Yunnan Province, China | Zhou.2016 |
| R.bie-2011A091D | 26.96 | 97.8 | 96.06 | SRR1041080<br>SRR1041081<br>SRR1041083<br>SRR1041084<br>SRR1041085<br>SRR1041086<br>SRR1041087<br>SRR1041089<br>SRR1041090 | *R. bieti* | Weixi, Yunnan Province, China | Zhou.2016 |
| R.bie-2011A092 | 11.55 | 97.25 | 99 | SRR1739994<br>SRR1739995<br>SRR1739996 | *R. bieti* | Weixi, Yunnan Province, China | Zhou.2016 |
| R.bie-2011A106 | 9.92 | 96.63 | 99.24 | SRR1739998<br>SRR1739999<br>SRR1740000<br>SRR1740001 | *R. bieti* | Weixi, Yunnan Province, China | Zhou.2016 |
| R.bie-2013A016 | 10.05 | 96.98 | 99.05 | SRR1740004<br>SRR1740005<br>SRR1740006<br>SRR1740007 | *R. bieti* | Weixi, Yunnan Province, China | Zhou.2016 |
| R.bie-2013A017 | 12.68 | 97.01 | 99.21 | SRR1740008<br>SRR1740009<br>SRR1740010<br>SRR1740011 | *R. bieti* | Weixi, Yunnan Province, China | Zhou.2016 |
| Rb0 | 51.23 | 98.0200 | 96.18 | SRR1300776<br>SRR1300778<br>SRR1300779<br>SRR1300780<br>SRR1300781<br>SRR1300782 | *R. bieti* | Weixi, Yunnan Province, China | Yu.2016 |
| Rb01 | 10.62 | 96.8700 | 98.47 | SRR1300622<br>SRR1300637 | *R. bieti* | Weixi, Yunnan Province, China | Yu.2016 |
| Rb02 | 10.2 | 96.7500 | 98.53 | SRR1300727<br>SRR1338686 | *R. bieti* | Weixi, Yunnan Province, China | Yu.2016 |
| Rb03 | 9.93 | 96.5900 | 98.42 | SRR1300739 | *R. bieti* | Weixi, Yunnan Province, China | Yu.2016 |

| | | | | | | | |
|---|---|---|---|---|---|---|---|
| | | | | SRR1338905 | | | |
| Rb04 | 11.3 | 96.9700 | 97.57 | SRR1300740 | *R. bieti* | Weixi, Yunnan Province, China | Yu.2016 |
| | | | | SRR1339085 | | | |
| Rb05 | 10.78 | 96.9600 | 98.17 | SRR1300741 | *R. bieti* | Weixi, Yunnan Province, China | Yu.2016 |
| Rb06 | 9.41 | 96.8500 | 98.6 | SRR1300742 | *R. bieti* | Weixi, Yunnan Province, China | Yu.2016 |
| | | | | SRR1339315 | | | |
| Rb07 | 9.77 | 96.8700 | 98.33 | SRR1300743 | *R. bieti* | Weixi, Yunnan Province, China | Yu.2016 |
| | | | | SRR1339453 | | | |
| Rb08 | 11.89 | 97.1000 | 97.92 | SRR1300744 | *R. bieti* | Weixi, Yunnan Province, China | Yu.2016 |
| | | | | SRR1339454 | | | |
| Rb09 | 10.96 | 96.8600 | 97.88 | SRR1300745 | *R. bieti* | Weixi, Yunnan Province, China | Yu.2016 |
| | | | | SRR1339455 | | | |
| Rb10 | 13.14 | 97.1900 | 96.64 | SRR1300747 | *R. bieti* | Weixi, Yunnan Province, China | Yu.2016 |
| | | | | SRR1339456 | | | |
| Rb11 | 10.93 | 97.0400 | 98.1 | SRR1300748 | *R. bieti* | Weixi, Yunnan Province, China | Yu.2016 |
| | | | | SRR1339457 | | | |
| Rb12 | 11.13 | 97.0000 | 98.4 | SRR1300750 | *R. bieti* | Weixi, Yunnan Province, China | Yu.2016 |
| | | | | SRR1339458 | | | |
| Rb13 | 10.6 | 96.9000 | 98.77 | SRR1300751 | *R. bieti* | Weixi, Yunnan Province, China | Yu.2016 |
| | | | | SRR1339459 | | | |
| | | | | SRR1339460 | | | |
| Rb14 | 11.81 | 96.8300 | 98.09 | SRR1300752 | *R. bieti* | Weixi, Yunnan Province, China | Yu.2016 |
| | | | | SRR1339461 | | | |
| Rb15 | 11.97 | 97.2800 | 97.66 | SRR2017644 | *R. bieti* | Weixi, Yunnan Province, China | Yu.2016 |
| | | | | SRR2017657 | | | |
| Rb16 | 13.37 | 97.3200 | 98.19 | SRR2017658 | *R. bieti* | Weixi, Yunnan Province, China | Yu.2016 |
| Rb18 | 10.93 | 96.9800 | 97.91 | SRR2017661 | *R. bieti* | Weixi, Yunnan Province, China | Yu.2016 |
| | | | | SRR2017659 | | | |
| Rb19 | 12.65 | 97.2700 | 98.16 | SRR2017663 | *R. bieti* | Weixi, Yunnan Province, China | Yu.2016 |
| R.bre-2011A082 | 9.17 | 97.13 | 98.87 | SRR1740012 | *R. brelichi* | Fanjing Mountaion, Guizhou Province, China | Zhou.2016 |
| | | | | SRR1740013 | | | |
| | | | | SRR1740014 | | | |
| R.bre-2011A084 | 10.64 | 97.38 | 99.06 | SRR1740015 | *R. brelichi* | Fanjing Mountaion, Guizhou Province, China | Zhou.2016 |
| | | | | SRR1740016 | | | |
| | | | | SRR1740017 | | | |
| R.bre-2011A104D | 9.89 | 96.73 | 99.09 | SRR1041069 | *R. brelichi* | Fanjing Mountaion, Guizhou Province, China | Zhou.2016 |
| | | | | SRR1041070 | | | |
| | | | | SRR1041071 | | | |
| | | | | SRR1041072 | | | |
| | | | | SRR1041073 | | | |
| | | | | SRR1041074 | | | |
| | | | | SRR1041075 | | | |
| | | | | SRR1041077 | | | |
| | | | | SRR1041078 | | | |
| | | | | SRR1041079 | | | |
| R.bre-2011A105 | 10.39 | 97.01 | 99.2 | SRR1740018 | *R. brelichi* | Fanjing Mountaion, Guizhou Province, China | Zhou.2016 |

| | | | | | | | |
|---|---|---|---|---|---|---|---|
| | | | | SRR1740019<br>SRR1740020<br>SRR1740021 | | | |
| R.bre6574 | 36.65 | 97.92 | 98.38 | SRR1588562 | *R. brelichi* | Fanjing Mountaion, Guizhou Province, China | Yu.2016 |
| R.rox_QLN-2013A002 | 8.84 | 97.38 | 98.56 | SRR1743029<br>SRR1743030<br>SRR1743031<br>SRR1743032 | *R. roxellana* (QL) | Qinling, Shaanxi Province, China | Zhou.2016 |
| R.rox_QLN-2013A014 | 9.06 | 97.52 | 99.19 | SRR1743033<br>SRR1743034<br>SRR1743035<br>SRR1743036 | *R. roxellana* (QL) | Qinling, Shaanxi Province, China | Zhou.2016 |
| R.rox_QLN-2013A015 | 9.45 | 97.74 | 99.16 | SRR1743037<br>SRR1743038<br>SRR1743039<br>SRR1743040 | *R. roxellana* (QL) | Qinling, Shaanxi Province, China | Zhou.2016 |
| R.rox_QLS-2011A062 | 10.01 | 96.39 | 99.3 | SRR1743041<br>SRR1743042<br>SRR1743043 | *R. roxellana* (QL) | Qinling, Shaanxi Province, China | Zhou.2016 |
| R.rox_QLS-2011A074 | 12.41 | 97.59 | 99.18 | SRR1743045<br>SRR1743046<br>SRR1743047<br>SRR1743048<br>SRR1743049 | *R. roxellana* (QL) | Qinling, Shaanxi Province, China | Zhou.2016 |
| R.rox_QLS-2011A093 | 12.41 | 97.59 | 99.18 | SRR1743053<br>SRR1743054<br>SRR1743055<br>SRR1743056 | *R. roxellana* (QL) | Qinling, Shaanxi Province, China | Zhou.2016 |
| SRR2017679 | 11.07 | 97.7500 | 94.39 | SRR2017679 | *R. roxellana* (QL) | Qinling, Shaanxi Province, China | Yu.2016 |
| SRR2017680 | 12.25 | 97.6800 | 99.36 | SRR2017680 | *R. roxellana* (QL) | Qinling, Shaanxi Province, China | Yu.2016 |
| SRR2017681 | 11.99 | 97.2900 | 99.21 | SRR2017681 | *R. roxellana* (QL) | Qinling, Shaanxi Province, China | Yu.2016 |
| SRR2017682 | 12.78 | 98.0300 | 99.23 | SRR2017682 | *R. roxellana* (QL) | Qinling, Shaanxi Province, China | Yu.2016 |
| SRR2017683 | 11.27 | 98.1200 | 99.27 | SRR2017683 | *R. roxellana* (QL) | Qinling, Shaanxi Province, China | Yu.2016 |
| SRR2017684 | 9.52 | 97.1300 | 99.00 | SRR2017684 | *R. roxellana* (QL) | Qinling, Shaanxi Province, China | Yu.2016 |
| SRR2017685 | 9.95 | 97.6400 | 99.23 | SRR2017685 | *R. roxellana* (QL) | Qinling, Shaanxi Province, China | Yu.2016 |
| SRR2017686 | 13.35 | 98.0400 | 97.76 | SRR2017686 | *R. roxellana* (QL) | Qinling, Shaanxi Province, China | Yu.2016 |
| SRR6942132 | 10.22 | 97.9500 | 97.80 | SRR6942132 | *R. roxellana* (QL) | Qinling, Shaanxi Province, China | Kuang.2019 |
| R.rox_GS-2011A047 | 12.23 | 97.54 | 99.31 | SRR1742924<br>SRR1742925<br>SRR1742926<br>SRR1742928<br>SRR1742929 | *R. roxellana* (SG) | Minshan, Gansu Province, China | Zhou.2016 |
| R.rox_GS-2011A049 | 7.56 | 97.02 | 99.27 | SRR1742930<br>SRR1742931<br>SRR1742932<br>SRR1742933<br>SRR1742934 | *R. roxellana* (SG) | Minshan, Gansu Province, China | Zhou.2016 |

| | | | | | | | |
|---|---|---|---|---|---|---|---|
| R.rox_GS-2011A052 | 27.95 | 85.27 | 99.15 | SRR1742935<br>SRR1742936 | *R. roxellana* (SG) | Minshan, Gansu Province, China | Zhou.2016 |
| R.rox_MS-2011A003 | 9.42 | 95.88 | 99.27 | SRR1742937<br>SRR1742938<br>SRR1742939 | *R. roxellana* (SG) | Minshan, Gansu Province, China | Zhou.2016 |
| R.rox_MS-2011A007 | 10.92 | 95.94 | 99.28 | SRR1742940<br>SRR1742941<br>SRR1742942 | *R. roxellana* (SG) | Minshan, Gansu Province, China | Zhou.2016 |
| R.rox_MS-2011A041 | 9.63 | 97.46 | 99.17 | SRR1742943<br>SRR1742944<br>SRR1742945<br>SRR1742946 | *R. roxellana* (SG) | Minshan, Gansu Province, China | Zhou.2016 |
| R.rox_MS-2012A003D | 14.2 | 97.97 | 89.73 | SRR1040961<br>SRR1040962<br>SRR1040963<br>SRR1040964<br>SRR1040965<br>SRR1040966<br>SRR1040967<br>SRR1040968<br>SRR1040969<br>SRR1040970 | *R. roxellana* (SG) | Minshan, Gansu Province, China | Zhou.2016 |
| R.rox_MS-2013A005 | 12.63 | 97.95 | 99.27 | SRR1742951<br>SRR1742953<br>SRR1742954<br>SRR1742956 | *R. roxellana* (SG) | Minshan, Gansu Province, China | Zhou.2016 |
| R.rox_MS-2013A006 | 11.24 | 97.62 | 99.25 | SRR1742966<br>SRR1742967<br>SRR1742968<br>SRR1742969 | *R. roxellana* (SG) | Minshan, Gansu Province, China | Zhou.2016 |
| R.rox_QLA-2012A005 | 11.6 | 97.64 | 99.31 | SRR1742958<br>SRR1742959<br>SRR1742961<br>SRR1742962<br>SRR1742964 | *R. roxellana* (SG) | Qionglai, Sichuan Province, China | Zhou.2016 |
| R.rox_QLA-2012A006 | 9.35 | 97.75 | 99.1 | SRR1742970<br>SRR1742971<br>SRR1742972<br>SRR1742973<br>SRR1742974 | *R. roxellana* (SG) | Qionglai, Sichuan Province, China | Zhou.2016 |
| R.rox_QLA-2012A007 | 9.5 | 97.77 | 99.09 | SRR1743018<br>SRR1743019<br>SRR1743020 | *R. roxellana* (SG) | Qionglai, Sichuan Province, China | Zhou.2016 |
| R.rox_QLA-2013A001 | 9.03 | 97.36 | 99.1 | SRR1743021<br>SRR1743022<br>SRR1743023<br>SRR1743024 | *R. roxellana* (SG) | Qionglai, Sichuan Province, China | Zhou.2016 |
| R.rox_QLA-2013A007 | 9.87 | 97.61 | 99.13 | SRR1743025<br>SRR1743026 | *R. roxellana* (SG) | Qionglai, Sichuan Province, China | Zhou.2016 |

| | | | | | | | |
|---|---|---|---|---|---|---|---|
| | | | | SRR1743027 | | | |
| | | | | SRR1743028 | | | |
| SRR2017664 | 12.23 | 98.4900 | 99.27 | SRR2017664 | *R. roxellana* (SG) | Qionglai, Sichuan Province, China | Yu.2016 |
| SRR2017665 | 11.39 | 98.0800 | 98.00 | SRR2017665 | *R. roxellana* (SG) | Minshan, Gansu Province, China | Yu.2016 |
| SRR2017666 | 13.68 | 98.2000 | 99.10 | SRR2017666 | *R. roxellana* (SG) | Minshan, Gansu Province, China | Yu.2016 |
| SRR2017667 | 11.67 | 97.6900 | 97.66 | SRR2017667 | *R. roxellana* (SG) | Minshan, Gansu Province, China | Yu.2016 |
| SRR2017668 | 12.48 | 97.9800 | 99.17 | SRR2017668 | *R. roxellana* (SG) | Minshan, Gansu Province, China | Yu.2016 |
| SRR2017669 | 12.67 | 97.7000 | 99.16 | SRR2017669 | *R. roxellana* (SG) | Minshan, Gansu Province, China | Yu.2016 |
| SRR2017670 | 9.51 | 97.7700 | 99.23 | SRR2017670 | *R. roxellana* (SG) | Minshan, Gansu Province, China | Yu.2016 |
| SRR2017671 | 13.33 | 97.7500 | 99.28 | SRR2017671 | *R. roxellana* (SG) | Minshan, Gansu Province, China | Yu.2016 |
| SRR2017672 | 10.91 | 97.4600 | 99.27 | SRR2017672 | *R. roxellana* (SG) | Minshan, Gansu Province, China | Yu.2016 |
| SRR2017673 | 10.78 | 97.7600 | 99.29 | SRR2017673 | *R. roxellana* (SG) | Minshan, Gansu Province, China | Yu.2016 |
| SRR2017676 | 12.66 | 97.5700 | 98.94 | SRR2017676 | *R. roxellana* (SG) | Minshan, Gansu Province, China | Yu.2016 |
| SRR6942131 | 11.18 | 97.8600 | 99.14 | SRR6942131 | *R. roxellana* (SG) | Minshan, Gansu Province, China | Kuang.2019 |
| R.rox_SNJ-2011A014 | 27.64 | 98.25 | 99.3 | SRR1743057 | *R. roxellana* (SNJ) | Shennongjia, Hubei Province, China | Zhou.2016 |
| | | | | SRR1743058 | | | |
| | | | | SRR1743059 | | | |
| R.rox_SNJ-2011A019 | 10.92 | 97.54 | 99.09 | SRR1743060 | *R. roxellana* (SNJ) | Shennongjia, Hubei Province, China | Zhou.2016 |
| | | | | SRR1743061 | | | |
| | | | | SRR1743062 | | | |
| | | | | SRR1743063 | | | |
| | | | | SRR1743064 | | | |
| R.rox_SNJ-2013A008 | 12.49 | 98.05 | 99.13 | SRR1743065 | *R. roxellana* (SNJ) | Shennongjia, Hubei Province, China | Zhou.2016 |
| | | | | SRR1743066 | | | |
| | | | | SRR1743067 | | | |
| | | | | SRR1743068 | | | |
| | | | | SRR1743069 | | | |
| R.rox_SNJ-2013A009 | 14.04 | 98.06 | 99.27 | SRR1743070 | *R. roxellana* (SNJ) | Shennongjia, Hubei Province, China | Zhou.2016 |
| | | | | SRR1743071 | | | |
| | | | | SRR1743072 | | | |
| | | | | SRR1743073 | | | |
| R.rox_SNJ-2013A011 | 12.32 | 97.64 | 99.32 | SRR1743074 | *R. roxellana* (SNJ) | Shennongjia, Hubei Province, China | Zhou.2016 |
| | | | | SRR1743076 | | | |
| | | | | SRR1743077 | | | |
| | | | | SRR1743078 | | | |
| R.rox_SNJ-2013A022 | 11.29 | 97.9 | 99.2 | SRR1743079 | *R. roxellana* (SNJ) | Shennongjia, Hubei Province, China | Zhou.2016 |
| | | | | SRR1743080 | | | |
| | | | | SRR1743081 | | | |
| RR22 | 10.69 | 97.5300 | 100.00 | SRR2017688 | *R. roxellana* (SNJ) | Shennongjia, Hubei Province, China | Yu.2016 |
| | | | | SRR2017689 | | | |
| RR23 | 10.62 | 97.8800 | 100.00 | SRR2017690 | *R. roxellana* (SNJ) | Shennongjia, Hubei Province, China | Yu.2016 |
| | | | | SRR2017692 | | | |
| SRR2017687 | 12.03 | 97.6500 | 98.74 | SRR2017687 | *R. roxellana* (SNJ) | Shennongjia, Hubei Province, China | Yu.2016 |
| SRR2017693 | 14.38 | 97.5700 | 99.33 | SRR2017693 | *R. roxellana* (SNJ) | Shennongjia, Hubei Province, China | Yu.2016 |

| | | | | | | | |
|---|---|---|---|---|---|---|---|
| SRR2017694 | 12.97 | 97.7800 | 97.54 | SRR2017694 | *R. roxellana* (SNJ) | Shennongjia, Hubei Province, China | Yu.2016 |
| SRR6942133 | 14.99 | 96.4700 | 98.12 | SRR6942133 | *R. roxellana*(SNJ) | Shennongjia, Hubei Province, China | Kuang.2019 |
| SRR6942134 | 13.92 | 97.6000 | 99.23 | SRR6942134 | *R. roxellana*(SNJ) | Shennongjia, Hubei Province, China | Kuang.2019 |
| R.str03076575 | 26.98 | 91.6 | 95.05 | SRR1588210 | *R. strykeri* | Gaoligong Mountain, Yunnan Province, China | Yu.2016 |
| | | | | SRR1588563 | | | |
| | | | | SRR1588566 | | | |
| R.str-2013A004D | 22.54 | 97.55 | 78.08 | SRR1041092 | *R. strykeri* | Gaoligong Mountain, Yunnan Province, China | Zhou.2016 |
| | | | | SRR1041093 | | | |
| | | | | SRR1041094 | | | |
| | | | | SRR1041095 | | | |
| | | | | SRR1041096 | | | |
| | | | | SRR1041098 | | | |
| R.str-2013A020 | 11.63 | 96.95 | 99.18 | SRR1743082 | *R. strykeri* | Gaoligong Mountain, Yunnan Province, China | Zhou.2017 |
| | | | | SRR1743083 | | | |
| | | | | SRR1743084 | | | |
| | | | | SRR1743085 | | | |
| R.str9021 | 15.85 | 97.2 | 98.63 | SRR13169613 | *R. strykeri* | Gaoligong Mountain, Yunnan Province, China | Kuang.2020 |
| R.str9022 | 19.4 | 97.5 | 99.2 | SRR13169622 | *R. strykeri* | Gaoligong Mountain, Yunnan Province, China | Kuang.2020 |
| R.str9023 | 19.4 | 97.5 | 98.55 | SRR13169621 | *R. strykeri* | Gaoligong Mountain, Yunnan Province, China | Kuang.2020 |
| R.avu-03076576 | 33.12 | 97.46 | 92.78 | SRR1588564 | *R.avunculus* | North Vietnam | Yu.2016 |
| | | | | SRR1588565 | | | |
| R.avu9020 | 9.91 | 90.87 | 95.05 | SRR13169614 | *R.avunculus* | North Vietnam | Kuang.2020 |
| SRR 10028097 | 31.15 | 94.2100 | 98.38 | SRR10028097 | *T. auratus* | | Liu.2019 |
| T.fra-2017B015 | 31.2 | 93.61 | 98.69 | SRR7778908 | *T. francoisi* | | |
| SRR 10028098 | 33.82 | 94.1100 | 98.83 | SRR10028098 | *T. laotum* | | |

**Table S2.** Distribution of the number of effects within various genomic regions of snub-nosed monkeys.

| Type (alphabetical order) | Count | Percentage |
|---|---|---|
| DOWNSTREAM | 3,601,122 | 5.81% |
| EXON | 381,169 | 0.62% |
| INTERGENIC | 10,400,067 | 16.79% |
| INTRON | 21,559,158 | 34.80% |
| SPLICE_SITE_ACCEPTOR | 509 | 0.00% |
| SPLICE_SITE_DONOR | 652 | 0.00% |
| SPLICE_SITE_REGION | 35,107 | 0.06% |
| TRANSCRIPT | 22,066,646 | 35.62% |
| UPSTREAM | 3,568,898 | 5.76% |
| UTR_3_PRIME | 267,342 | 0.43% |
| UTR_5_PRIME | 78,573 | 0.13% |

**Table S3.** Combinations of individuals with estimated kinship coefficients over 0.354 from the results of kinship analyses, these pairs of individuals considered to be duplicate or MZ twin.

| ID1 | ID2 | N_SNP | Z0 | Phi | HetHet | IBS0 | Kinship |
|---|---|---|---|---|---|---|---|
| R.rox_QLS-2011A074 | R.rox_QLS-2011A093 | 17341236 | 1 | 0 | 0.061 | 0 | 0.4999 |
| R.rox_SNJ-2013A008 | R.rox_SNJ-2013A022 | 17300958 | 1 | 0 | 0.062 | 0.0001 | 0.4529 |
| SRR2017694 | SRR6942133 | 17052507 | 1 | 0 | 0.064 | 0.0004 | 0.4485 |
| R.bie-2011A091 | R.bie-2011A091D | 16515172 | 1 | 0 | 0.055 | 0.0002 | 0.4282 |
| R.bre-2011A104D | R.bre6574 | 17183434 | 1 | 0 | 0.029 | 0.0001 | 0.4196 |
| Rb15 | Rb18 | 17058132 | 1 | 0 | 0.051 | 0.0006 | 0.3923 |
| R.str-2013A020 | R.str03076575 | 17308198 | 1 | 0 | 0.023 | 0.0001 | 0.3811 |
| Rb16 | Rb19 | 16816379 | 1 | 0 | 0.056 | 0.0005 | 0.3722 |

**Table S4.** Using the ABBA-BABA analysis to calculate gene flow for potential admixed individuals (P3) filtered out through phylogenetic results.

| P1 | P2 | P3 | Dstatistic | Z-score | p-value | f4-ratio | BBAA | ABBA | BABA |
|---|---|---|---|---|---|---|---|---|---|
| QL.R.rox_QLN-2013A002 | QL.SRR6942132 | SG.SRR2017665 | 0.128258 | 7.28297 | 3.27e-13 | 0.168155 | 322168 | 266970 | 206273 |
| QL.R.rox_QLS-2011A062 | QL.SRR6942132 | SG.SRR2017666 | 0.112779 | 7.58176 | 3.41e-14 | 0.127873 | 316208 | 254740 | 203105 |
| QL.R.rox_QLS-2011A093 | QL.SRR2017685 | SG.SRR2017667 | 0.0779779 | 4.98212 | 6.29e-07 | 0.0845474 | 350125 | 236525 | 202306 |
| QL.R.rox_QLS-2011A062 | QL.SRR6942132 | SG.SRR2017673 | 0.089487 | 7.02096 | 2.20e-12 | 0.10163 | 317995 | 249615 | 208610 |
| QL.R.rox_QLS-2011A062 | QL.SRR6942132 | SG.SRR2017676 | 0.0973666 | 6.71717 | 1.85e-11 | 0.117985 | 323708 | 250057 | 205683 |
| QL.R.rox_QLN-2013A002 | QL.SRR2017680 | SG.SRR2017664 | 0.0815991 | 4.17165 | 3.02e-05 | 0.0550434 | 469863 | 178634 | 151681 |
| QL.SRR2017685 | QL.R.rox_QLS-2011A093 | SG.R.rox_QLA-2012A005 | 0.126108 | 5.46881 | 4.53e-08 | 0.10465 | 345221 | 250842 | 194661 |
| QL.SRR2017680 | QL.R.rox_QLN-2013A002 | SG.R.rox_QLA-2012A007 | 0.130011 | 5.74309 | 9.30e-09 | 0.0781789 | 491158 | 181140 | 139459 |
| SG.SRR2017672 | SG.R.rox_GS-2011A047 | QL.R.rox_QLN-2013A014 | 0.211714 | 8.09826 | 5.57e-16 | 0.1627 | 476624 | 224252 | 145888 |
| SG.SRR2017672 | SG.R.rox_GS-2011A047 | QL.R.rox_QLN-2013A015 | 0.214253 | 7.91191 | 2.53e-15 | 0.169715 | 473378 | 226149 | 146342 |

*Note.* significant gene flow (Z-score >3)
